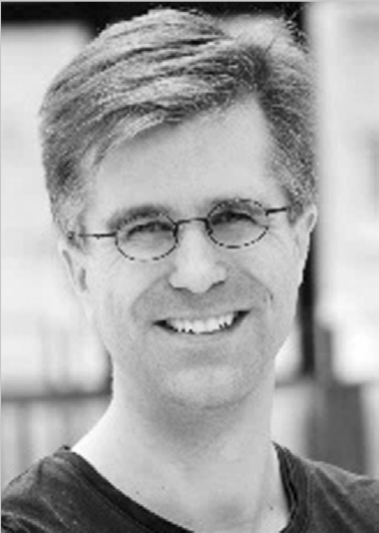# It's in the Fine Print: Erasable Three‐Dimensional Laser‐Printed Micro‐ and Nanostructures

**DOI:** 10.1002/anie.201910634

**Published:** 2020-01-23

**Authors:** David Gräfe, Sarah L. Walden, James Blinco, Martin Wegener, Eva Blasco, Christopher Barner‐Kowollik

**Affiliations:** ^1^ Centre for Materials Science, School of Chemistry and Physics Queensland University of Technology (QUT) 2 George Street QLD 4000 Brisbane Australia; ^2^ Macromolecular Architectures Institute for Technical Chemistry and Polymer Chemistry (ITCP) Karlsruhe Institute of Technology (KIT) Engesserstrasse 18 76131 Karlsruhe Germany; ^3^ Institute of Applied Physics (APH) Karlsruhe Institute of Technology (KIT) Engesserstrasse 18 76131 Karlsruhe Germany; ^4^ Institute of Nanotechnology (INT) Karlsruhe Institute of Technology (KIT) Hermann-von-Helmholtz-Platz 1 76344 Eggenstein-Leopoldshafen Germany

**Keywords:** 3D printing, degradable materials, direct laser writing, labile bonds, photochemistry

## Abstract

3D printing, on all scales, is currently a vibrant topic in scientific and industrial research as it has enormous potential to radically change manufacturing. Owing to the inherent nature of the manufacturing process, 3D printed structures may require additional material to structurally support complex features. Such support material must be removed after printing—sometimes termed subtractive manufacturing—without adversely affecting the remaining structure. An elegant solution is the use of photoresists containing labile bonds that allow for controlled cleavage with specific triggers. Herein, we explore state‐of‐the‐art cleavable photoresists for 3D direct laser writing, as well as their potential to combine additive and subtractive manufacturing in a hybrid technology. We discuss photoresist design, feature resolution, cleavage properties, and current limitations of selected examples. Furthermore, we share our perspective on possible labile bonds, and their corresponding cleavage trigger, which we believe will have a critical impact on future applications and expand the toolbox of available cleavable photoresists.

## Graphical abstract

**Write on**: State‐of‐the‐art cleavable photoresists for 3D direct laser writing, particularly photoresist design, feature resolution, and cleavage properties of selected examples are presented. Furthermore, possible labile bonds, which will have a critical impact on future applications are discussed.

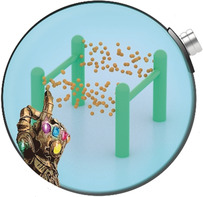

## 1 Introduction

Three‐dimensional (3D) printing is an additive manufacturing process developed in the early 1980s that translates a computer‐aided design into a physical object.^5^ In recent years, 3D printing has attracted much interest in the industrial and scientific community as it offers enormous potential to radically transform current manufacturing approaches. It is estimated that by 2030, the market share of goods produced by additive manufacturing could be over 10 % in all sectors.^6^ For this reason, 3D printing, as an intelligent processing technique, is also seen as an essential ingredient in the era of industry 4.0—also referred to as “Fourth Industrial Revolution”.^7^ The main advantages of 3D printing over traditional manufacturing techniques are:

1. rapid and low‐cost production of prototypes and low‐demand goods
2. separation of production from product design
3. ecological production through optimal use of materials and reduction of waste volume
4. simple production of complex geometries with customized form factors (e.g. size and shape).^5^

These advantages, combined with falling prices for additive manufacturing technologies, have enabled implementation in industry, laboratories, and even private households.

By far the largest and most attractive market segment for 3D printing is photopolymerization.^8^ Additive manufacturing via photopolymerization involves solidifying a liquid resin into a 3D object by linking monomers together to form a polymer network. By spatially controlling the reaction using a tightly focused light source, light‐based 3D printing techniques can offer the highest resolution currently on the market.^9^ There is a variety of different light‐based 3D printing techniques available including stereolithography,^10^ continuous liquid interface production,^11^ digital light processing,^12^ direct laser writing (DLW),^13^ and many others.^8^, ^9^ Each technique has its own advantages and disadvantages regarding printing speed, minimum accessible voxel size, costs, and the integration of different materials. Space limitations do not permit us to discuss all of these techniques in detail. Therefore, herein we will focus exclusively on DLW, which provides the highest lateral resolution of all light‐based 3D printing techniques. There are excellent Reviews which cover advances in various other areas of light‐based 3D printing research.^8^, ^9^

In the early days of DLW research, the main focus was on developing technologies that enabled higher printing speeds, larger build volumes, and improved spatial resolution. Functionality of the manufactured materials is typically achieved through the well‐defined 3D forms with precise spatial orientation that DLW allows.^14^ However, most applications require responsive and/or adaptive materials that exhibit disparate chemical, electrical, mechanical, optical or thermal properties to achieve the desired function. A field of research is emerging that focuses on cleavable materials which can be removed in a controlled manner after printing. Although there is no consensus on the definition, controlled removal utilizing cleavable photoresists is now often referred to as subtractive manufacturing to distinguish it from additive manufacturing. Thus, DLW with cleavable photoresists combines the virtues and possibilities of additive and subtractive manufacturing and opens up new opportunities by offering more design freedom, flexibility, and intricacy in fabricating complex components. In the optimal case, the advantages of DLW and cleavable photoresists would be exploited synergistically, and their inherent limitations and drawbacks be minimized.

In this Minireview, we discuss the molecular design, application, and unrealized potential of cleavable photoresists for DLW. First, we briefly introduce DLW with an emphasis on non‐cleavable photoresists and the resulting material properties. The major part of the Minireview covers existing photoresists with labile bonds that are cleavable by specific triggers such as light, additives, or enzymes. Note that decomposition processes, such as plasma etching and pyrolysis, which provide little control over the cleavage process, are beyond the scope of this Minireview, but there are several examples that can be found elsewhere.^15^ Finally, we share our vision for the future of cleavable photoresists in DLW which we believe will have significant impact in adaptive 3D micro‐ and nanoprinting for future applications in cell biology, microfluidics, and micro‐optics.

## 2 Fundamentals of Direct Laser Writing

DLW, also known as direct laser lithography, 3D laser lithography or 3D laser printing, was introduced by Maruo in 1997 and offers by far the highest resolution of any light‐based 3D printing technique.^13^

The basic principle of DLW is to polymerize a liquid resist only in the region close to the most intense spot of a focused laser beam. This is most commonly achieved by a nonlinear optical process known as two‐photon absorption, where two low‐energy photons are simultaneously incident on a molecule, collectively providing sufficient energy to be absorbed. The likelihood of two photons arriving simultaneously is extremely small at low intensities but increases quadratically as the intensity increases. Therefore, absorption, and hence polymerization, is localised only to the most intense region of a focused laser where the photon flux is high. This three‐dimensional localised region where printing occurs is known as a voxel, the volume element named in analogy to the familiar 2D picture element or pixel.

The minimum feature size in DLW is not limited by the wavelength of light.^16^ In contrast, the minimum separation between two adjacent features, the resolution, is limited by the wavelength. This limit is often referred to as the Abbe diffraction barrier. However, a closer inspection yields that this limit is rather given by the two‐photon Sparrow criterion. For oil‐immersion focusing conditions, it is roughly given by one quarter of the free‐space wavelength of the light in the lateral direction, and by at least twice that value in the axial direction.^16^

It follows then that to improve the resolution as much as possible, short wavelength lasers focused with a high numerical aperture focusing lens are desired. However, the laser wavelength must be selected to suit the (two‐photon) absorption spectra of the photoinitiator. This has led to the common use of femtosecond pulsed frequency‐doubled near‐infrared fibre lasers; although printing has also been demonstrated with much more readily available 405 and 532 nm continuous‐wave lasers.^17^

**Figure 1:**
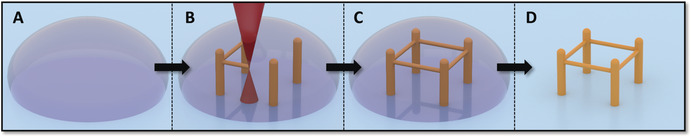
Typical procedure for fabricating arbitrary 3D microstructures with DLW and a liquid photoresist: A) Drop‐casting the negative‐tone photoresist onto a glass coverslip. B),C) Fabricating the 3D structure by moving the focused femtosecond laser through the photoresist. D) Final 3D structure after removal of the remaining non‐crosslinked photoresist.

A typical photoresist used in DLW consists of a two‐photon initiator and monomer(s), having multiple polymerizable end‐groups. By placing the liquid photoresist on a piezoelectric stage, which moves relative to the laser focal point, arbitrary 3D microstructures, with feature resolutions down to the submicron range (around 100 nm), can be successively built up. After printing, the 3D structure is developed by immersing the sample in a suitable solvent to remove the remaining material that is not sufficiently crosslinked. As a result, the 3D structure remains on the substrate as a replica of the photo pattern (Figure 1). Plainly speaking, DLW is like writing in 3D with a pen of light.

## 3 Cleavable Photoresists for Direct Laser Writing

Many factors influence network formation and the associated material properties of the final polymer product. For example, entrapment of pendant double bonds and unreacted monomers can limit effective crosslinking efficiency, resulting in a mechanically unstable material. In addition, polymerization temperature, the chemical nature of the crosslink, steric effects, and number of polymerizable units per molecule have been shown to influence the formation of networks. This is particularly relevant for the last variable whereby the number density of crosslinks directly correlates with the mechanical strength of a network. If equal weight fractions are used, a trifunctional crosslinker generally forms harder materials more rapidly than the equivalent bifunctional analogue.^18^ For this reason, most reported macroscopic networks, such as hydrogels, are based on bifunctional crosslinkers (e.g. di(meth)acrylates),^19^ while DLW applications often require tri‐ or tetrafunctional crosslinkers (e.g. pentaerythritol triacrylate (PETA)) to fabricate structures with fine details.^2^, ^3^, ^4^, ^20^

Similar to other photolithography techniques, common resins for DLW consist of epoxy and (meth)acrylate monomers. These resins, which also include a suitable two‐photon initiator, typically produce materials with low shrinkage and favourable mechanical properties.^21^ However, materials fabricated with these resins are irreversibly crosslinked into a permanent, unalterable shape without smart properties such as adaptability, responsiveness, or reconfigurability. To unleash the vast potential of DLW (and 3D printing in general), it is necessary to design advanced photoresists which have these intrinsically programmed properties. In doing so, the potential applications of DLW micro‐ and nano‐sized objects can be extended into new fields.

### 3.1 Applications of Cleavable Microstructures

A common limitation of 3D printing is that complex geometries need to incorporate sacrificial material to provide structural support. For example, support material is required under some largely overhanging features, to minimize distortion in hollow areas and to allow multiple moving components in the same part. Support material increases the build time and must be removed after the completion of the print. Unfortunately, physically fracturing off the support material from the object often has an adverse effect on the surface finish and leaves visual defects, not to mention the practical challenges in physically removing small portions of 3D printed objects on the micro‐ and nano‐scale.^22^ One solution to this problem lies in the incorporation of photoresists containing labile bonds or linkages. The cleavage of labile linkages upon specific external triggers allow the crosslinked 3D structure to disassociate into soluble fragments, which in turn can be washed away with suitable solvents.

In addition to their use as support structures, cleavable photoresists open new avenues of research in cell biology, where laser‐printed structures can be used as microscaffolds for cell positioning.^23^ In this application, the locally controlled, or complete cleavage of cell microscaffolds has the potential to provide insights into the response of cells to environmental changes as well as the mechanisms underlying adaptive processes.^24^ An alternative future application for cleavable photoresists is microfluidics. It is predicted that 3D printing will replace most current manufacturing processes in academia for microfluidic devices, including moulding of poly(dimethylsiloxane) and plastic, as alignment problems occur with multiple layers and microchannels cannot be made with arbitrary aspect ratios.^25^ Since cleavable valves and bridges are often used for automated fluid handling,^26^ photoresists containing labile bonds would accelerate innovation in this field. Finally, cleavable photoresists open up new possibilities for inorganic materials that use DLW structures as templates for complex, but well‐controlled architectures.^27^ Conventional etching methods require somewhat harsh conditions and typically suffer from incomplete template removal and cavity distortion. Furthermore, they are usually not selective. Cleavable photoresists with labile moieties can offer a gentler and more specific alternative to drive future innovations in that research area.

### 3.2 Temporal and Spatial Control of the Cleavage Process

**Figure 2:**
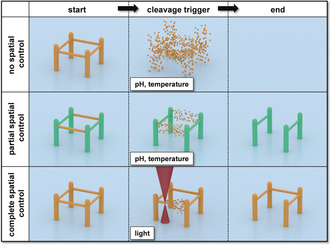
Possible avenues to spatially control the cleavage of 3D printed microstructures. Top: cleaving a single‐material 3D structure with a homogeneous external condition, such as pH, temperature, or a specific additive. Middle: Cleaving a multi‐material 3D structure made of cleavable and non‐cleavable features with a homogeneous external condition. Bottom: Cleaving a single‐material 3D structure with a focused light source.

A particular challenge in DLW with cleavable photoresists is achieving temporal and spatial control over the cleavage process. With regard to spatial control, cleaving a material can be divided into three categories: 1) no spatial control, 2) partial spatial control, and 3) complete spatial control (Figure 2). No spatial control (category 1) is the easiest to achieve and requires a photoresist that can be degraded by a homogeneous external condition, such as pH, temperature,^1^ specific additives,^3^ or enzymes.^4^, 20b Complete cleavage without spatial control is particularly interesting, for example in drug delivery, when the cargo is only transported by a direct‐laser‐written structure and cleavage is used for controlled cargo release.^4^, 20b Structures made of different materials provide an approach for partial spatial control of the cleavage process. One material of a multi‐material structure can be degraded by a specific stimulus, while the other material remains unaffected.^2^, 20a However, fabrication of a multi‐material structure with DLW is rather a time‐consuming process as it requires multiple photoresists and fabrication steps, as well an extra layer of complexity owing to realigning the resin for each step on such a small scale. An unexplored way to overcome these issues could be to use a multi‐material photoresist that can be orthogonally cured by disparate colours of light, allowing single‐step multi‐material fabrication as recently reported for single‐photon polymerization.^28^ Finally, complete spatial control of the cleavage could be accomplished by photolithographic techniques, such as masks or focused light, which stimulate cleavage only in a specific area. The ability to completely spatially control the degradation greatly expands DLW capabilities and increases the number of materials that can be fabricated readily (Table 1).

**Table 1:** Labile crosslinkers used for degradable microstructures fabricated with DLW.

Trigger	Labile Linkage	Cleavage Conditions	Cleavage Time	Feature Resolution	Reference
Temperature	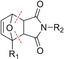	105 °C in furfuryl alcohol	–	–	1
Reduction	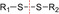	Dithiothreitol at 50 °C	15 min	300 nm	2
Additive	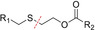	1 m ethanolamine at 50 °C in DMF	20 h	–	20a
	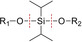	Inorganic salts in MeOH at room temperature or 50 °C	Between 15 min and 2 h	165 nm	3
Enzyme	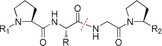	Collagenase type II	3 min at 0.5 mg mL^−1^ enzyme	–	4
		Matrix metalloproteinase‐2	5 h at 4 mg mL^−1^ enzyme; 118 h at physiological enzyme concentration	Diameter of microswimmers 6 μm	20b
Light	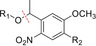	700 nm laser	Immediate cleavage	–	20c

In addition to spatial control, temporal control is necessary to dictate when the cleavage will commence. This ability is critical for fabricated materials that must be shape‐retaining as a temporary support function. Although fabrication parameters, such as laser power and writing speed, play a role in the stability and therefore cleavability of a material,^29^ temporal control of the cleavage is mainly determined by the synthetic design of the photoresist, thus providing considerable opportunities for novel materials. To control the stability and cleavability of 3D printed materials, the corresponding photoresist requires labile bonds or linkages, which can be decomposed on‐demand with a specific trigger. It is worth noting that an important prerequisite for labile bonds in DLW applications is that they must be relatively insensitive to free‐radical attack to avoid side reactions during the DLW fabrication process. A large body of work has been established that extensively exploits the controlled cleavage of networks via labile moieties, such as gels,^19^ solid‐phase organic synthesis,^30^ drug‐delivery systems,^31^ and biodegradable polymers,^32^ among others.^33^ However, research regarding cleavable 3D printed materials fabricated in the submicron range is still challenging and only a few examples have been reported so far, which will be discussed in the next Section.^2^, ^3^, ^4^, 20a, 20b Consequently, the translation of existing knowledge for the next generation of DLW photoresists is of significant value for the design of degradable micro‐ and nano‐sized printed 3D materials.

### 3.3 Temperature‐Labile Photoresists

**Figure 3:**
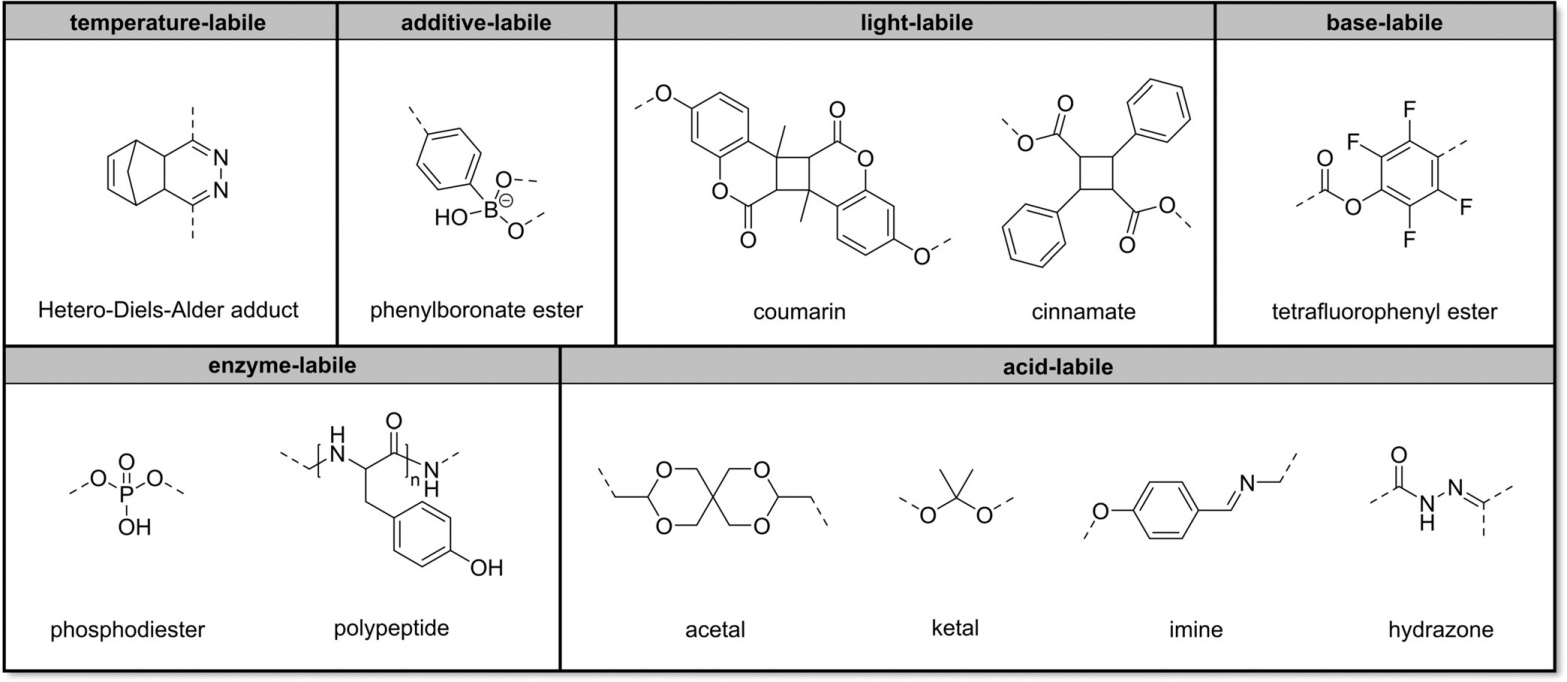
Selection of labile linkages that have potential in fabrication of cleavable microstructures via DLW.

**Scheme 1:**
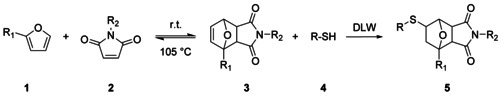
Mechanism for the 3D photofixation ligation and network formation using a DA and a thiol‐ene reaction.^1^

The Diels–Alder (DA) cycloaddition between a 1,3‐diene and a dienophile has been frequently utilized in the preparation of thermally responsive materials because of the efficient nature of the DA adduct formation and cleavage. Typically, the DA adduct is formed at ambient temperature, but also dissociates above the cycloreversion temperature, which is termed retro‐DA.^34^ Pioneering work by Adzima et al. in 2012 first demonstrated the use of cleavable photoresists based on DA adducts.^1^ Their photoresist contained a stoichiometric mixture of tetrakis furfuryl thiopropionate, 1,13‐bismaleimido 4,7,10‐trioxatridecane, and pentaerythritol tetrakis‐3‐mercaptopropionate, as well as Irgacure 184 as a photoinitiator. In a photofixation step, multifunctional furan **1** and maleimide **2** monomers form an oxy‐norbornene species **3** by a DA reaction (Scheme 1). While irradiating the fixated photoresist, the oxy‐norbornene species **3** undergoes a thiol‐ene reaction with the multifunctional thiol monomers **4** to form an irreversible crosslinked network **5** that forms the final 3D printed structure. To remove unexposed material and isolate the 3D printed structure, the sample was subsequently heated to 105 °C in furfuryl alcohol, triggering the retro‐DA reaction. As the cleavable photoresist is only used to fix the final 3D printed microstructure, there is no information available on feature resolution or the heating time required for cleavage, both of which are critical for evaluating the performance of the photoresist. In addition, the elevated cleavage temperature will certainly limit the practical utility of this photoresist. Given the numerous studies on DA adducts with a wide range of accessible cycloreversion temperatures, further research effort is required to design photoresists with predictable and practically relevant cleavage properties. In particular, the incorporation of heteroatoms into the DA adduct structure constitutes a challenge, but is very attractive for obtaining cleavable materials in the desired temperature window (Figure 3).^34^ Already, retro‐DA reactions have been triggered using high‐powered CO_2_ lasers, demonstrating the potential of this approach to provide complete spatial control over the cleavage.^35^

### 3.4 Redox‐labile Photoresists

**Figure 4:**
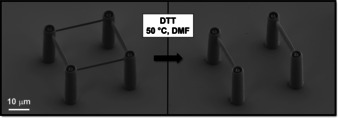
Scanning electron microscopy (SEM) images showing the partial spatial control of the degradation of a multi‐material box‐ring structure reported by Zieger et al.^2^ Only features written with the cleavable photoresist dissolve in a DTT solution at 50 °C, while features written with a commercially available photoresist remain intact. Adapted from Ref. 2.

Disulfide‐based materials capable of controlled degradation are widely used in various research fields including chemosensors,^36^ drug delivery,^37^ tumour imaging,^38^ and many others.^30^, ^37^ A well‐established approach to disulfide bond cleavage is the addition of an excess reducing agent, such as 2‐mercaptoethanol or dithiothreitol (DTT), which initiates the efficient and rapid thiol‐disulfide exchange. Some of us have demonstrated partial spatial control over cleavage using a photoresist consisting of commercial pentaerythritol tetrakis(mercaptoacetate), a synthesized phenacyl sulfide linker and tetrahydrothiophene‐1,1‐dioxide as solvent.^2^ After laser exposure, the photoresist forms disulfide bonds in a free step‐growth polymerization via reactive thioaldehydes without the requirement of an additional photoinitiator. Various model structures, such as woodpiles, blocks, and lines with a lateral resolution down to 300 nm, were successfully fabricated. Using this photoresist, partial spatial control over the cleavage was shown using multi‐material box‐ring structures containing both non‐cleavable and cleavable features. Features written with the cleavable photoresist could be selectively removed with a solution of DTT at 50 °C within 15 min, while features consisting of the non‐cleavable photoresist remained unaffected by the cleavage reagent (Figure 4). One shortcoming of this approach is the necessity for somewhat elevated temperatures for the complete removal of the cleavable photoresists. While this is not an issue for many applications, milder cleavage procedures are particularly valuable for clinical applications that require cleavage under physiological conditions.

### 3.5 Additive‐Labile Photoresists

Another study by Zieger et al. demonstrated similar spatial control employing a photoresist for micro‐ and macroscopic 3D printed structures suitable for standard stereolithography and DLW.20a The main components of the photoresists are a multifunctional thiol and a bifunctional acrylate in conjugation with a photoinitiator, which rapidly forms a sulfide‐based network after irradiation with a light source. The photoresist formulation also contains pyrogallol and vinylphosphonic acid as stabilizers to prevent self‐initiated radical thiol‐ene conjugation. Notably, all components of the photoresist formulation are commercially available, meaning the photoresist formulation can be easily adapted to the printing techniques used. Complex macro‐ and micro‐scale 3D structures with overhanging features requiring support material have been successfully fabricated. Complete removal of supporting material by aminolysis with an ethanolamine solution at 50 °C was visualized via optical time‐lapse microscopy and film records.

**Figure 5:**
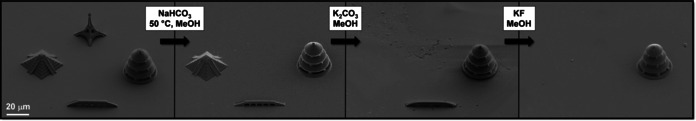
SEM images showing the sequential cleavage of silane‐based microstructures with methanol solution containing NaHCO_3_, K_2_CO_3_, or KF (scale bar=20 μm). Only the 3D structure (Asian temple) made of pure PETA remained unaffected. Adapted from Ref. 3.

A major challenge for materials with several removable parts is orthogonality, which makes it possible to selectively remove parts in the presence of others in any controlled order. Because microstructures fabricated via DLW can be used as templates,^27^ orthogonal cleavable materials enable the design of new materials that are currently inaccessible. Some of us recently reported a cleavable photoresist system based on labile silane crosslinkers that allow the chemoselective degradation of multiple 3D printed microstructures under mild conditions.^3^ Three crosslinkers containing methyl, ethyl, or isopropyl substituents on the silicon atom were mixed with a two‐photon photoinitiator and PETA. PETA was added to the photoresist formulation to enhance the direct laser written structures with regard to their feature resolution allowing resolutions down to 165 nm. For the targeted cleavage of the fabricated 3D microstructures, a simple methanol solution containing inorganic salts is required (Figure 5). Complete removal, independent of the applied photoresists, was observed within 2 h via optical microscopy and SEM. Note that our set of silane‐based photoresists requires a specific order and does not yet match the definition of true orthogonality. To enable truly orthogonally cleavable microstructures, we consider it necessary to combine different external stimuli for our cleavage triggers, such as pH and light, or substrate‐specific enzymes, which do not interfere with each other.

Another promising motif for an additive‐induced cleavage is phenylboronic acid (Figure 3).^39^ The primary advantage of phenylboronic acid is the unique ability to reversibly bind to 1,2‐diol or 1,3‐diol, allowing an added saccharide, such as glucose, to initiate a catalyst‐free dynamic exchange reaction at ambient conditions. Using glucose as an exchange molecule is particularly interesting for biomedical applications, as it is a primary source of energy for most living organisms. While there are promising advances in this area, there are still obstacles to biomedical applications that need to be considered when developing cleavable phenylboronic acid‐based photoresists. The phenylboronate ester formation is pH dependent and requires a pH value at or above the p*K*_A_ of the phenylboronic acid. As most phenylboronic acids have significantly high p*K*_A_ values, outside of what is practical for biomedical applications, strategies such as the incorporation of amine groups would be necessary to reduce the p*K*_A_ value into a more biologically accessible regime.^40^

### 3.6 Enzyme‐Labile Photoresists

Recently, the use of enzyme‐cleavable materials for biological and clinical applications has had caught attention, owing to the excellent biocompatibility of enzymes with minimal undesirable side effects being observed in humans.^41^ In addition to their remarkably good safety profile, enzymes often have a high substrate specificity limited to one amino acid or to a sequence of amino acids. For this reason, enzyme cleavable linkers have been employed for targeted drug release as they increase the tissue‐specific accumulation and penetration of the drug.^42^ Besides the actively targeted drug release, precise control over the shape and dimension of a drug delivery system can improve the drug efficacy and positively affect the therapeutic outcome.^43^ Therefore, the union of enzyme‐cleavable materials and DLW is an attractive approach to produce the next generation of drug delivery systems.

**Figure 6:**
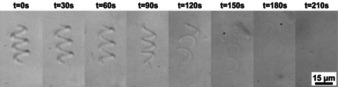
Optical microscope images showing the degradation of a hydrogel microswimmer based on gelatin methacryloyl in a collagenase solution (0.1 mg mL^−1^). Adapted from Ref. 4.

Wang et al. and Ceylan et al. fabricated hydrogel microswimmers based on gelatin methacryloyl (Figure 6).^4^, 20b Their magnetically actuated microswimmers, with dimensions in the micron‐range (6 μm in diameter by Ceylan et al.), provide 3D motion capability in liquid environments and allow for targeted drug delivery. Complete cleavage of their hydrogel structures without toxic residues in the presence of type II collagenase or matrix metalloproteinase‐2 enzymes was demonstrated within a clinically relevant time frame of minutes to hours (Figure 6).

However, hydrogels tend to be mechanically weak, which limits their practical application in biological systems, for example, as cell scaffolds. Approaches, such as double‐network hydrogels, ionic coordination interactions, filler materials, and many others, have been reported to improve the mechanical strength and fatigue resistance of polymer networks.^44^ Consequently, the focus should now be on developing enzyme cleavable materials with tailored mechanical properties, such as high mechanical strength combined with elasticity.

In addition to improving their mechanical properties, other enzyme‐cleavable linkages should be adopted for DLW applications. Linkages based on phosphodiesters, which are cleavable with phosphatase enzymes, have been introduced for solid‐phase peptide synthesis (Figure 3).^45^ Another interesting enzyme for the targeted cleavage of labile linkages is chymotrypsin. Chymotrypsin is a digestive enzyme within pancreatic juice that is capable of cleaving proteins and polypeptides based on large hydrophobic amino acids, such as tyrosine, tryptophan, and phenylalanine.42a Polypeptides consisting solely of repeated hydrophobic amino acids with multiple polymerizable end‐groups could be synthesized (Figure 3). This will be challenging, owing to the need for protective group chemistry, however the benefits of developing a polypeptide‐based photoresist would be the inherent biocompatibility and biological activity associated with an ideal profile for drug and gene delivery applications.

### 3.7 Photo‐Labile Photoresists

Spatially controlled degradation by utilizing photo‐labile crosslinkers remains a current challenge of 3D laser lithography. The scientific challenge in creating a photo‐labile photoresist is that the absorption of the photoinitiator and the photo‐labile crosslinker must not overlap, allowing for radical formation via two‐photon absorption without simultaneously degrading the photo‐labile bond. There are many interesting motifs that are worth exploring to create photo‐labile resists (Figure 3). Coumarin and cinnamate derivatives, for example, are often used as photo‐labile groups.^46^ However, the efficiency of the photoinduced cleavage is typically moderate to low and requires sophisticated substitution strategies of the leaving group to improve the photocleavage.

Some of us recently reported a breakthrough in designing a photo‐labile photoresist enabling additive fabrication of 3D microstructures using one wavelength and subsequent spatially controlled cleavage of the printed resist using another wavelength.20c The photoresist is composed of a bifunctional acrylate crosslinker containing a photo‐labile *o*‐nitrobenzyl ether moiety. 3D microstructures are written by photoinduced radical polymerization, using Ivocerin as the photoinitiator, upon exposure to a 900 nm laser. Subsequent scanning using a 700 nm laser allows for the selective removal of the cured resist by photocleaving the *o*‐nitrobenzyl group. Highly defined features, such as sub‐micrometric channels and text were cleaved inside the structures and imaged using SEM and laser scanning microscopy. In addition, a single wire bond was successfully eliminated from an array proving the possibility of complete partial removal of structures on‐demand.

We are convinced that one of the most exciting triggers to cleave a material is darkness. Some of us recently pioneered light‐stabilised dynamic materials (LSDM) based on naphthalenes and triazolinediones that require green LED light to stabilise the crosslinked material.^47^ Once the LED light is off the material, the chemical bonds of the network structure break up and the material becomes soft and liquefies. It would be a breakthrough if LSDMs could be utilized as dynamic photoresist for DLW applications. We may envisage a scenario where LSDM are used as a cell scaffold or drug delivery system and switching off the light releases the cargo. Similarly, LSDM‐based support material could stabilize critical features of a complex object in situ during the printing process, and after the critical feature has been printed, the LED light is turned off and the support material liquefies again.

### 3.8 pH‐Labile Photoresists

A further important trigger that is applicable to cleaving labile linkages is the pH value. Acid‐triggered cleavage has attracted interest in clinical applications, such as drug delivery research and tissue engineering, as the cellular conditions within a tumour or inflammatory tissue (pH value between 4.5 and 6.5) are very different from those in blood and normal healthy tissue (pH value around 7.4).^19^ This pH gradient allows for site‐specific drug deposition without prior knowledge of the target tissue site. Similarly, direct‐laser‐written structures with the ability to slowly degrade under physiological conditions may also be desirable for a time‐controlled drug delivery. Despite this attractive potential for the controlled drug delivery, to date there has been no report of a pH‐cleavable direct‐laser‐written structure. This yet unexplored topic of pH‐cleavable photoresists offers a new design space for chemists.

Figure 3 shows a selection of promising pH‐labile linkages that we believe are suitable for degradable microstructures. For instance, acetals are generally known to be pH‐labile linkages that can easily be introduced into a crosslinker structure, as they do not affect radically induced polymerization and can be hydrolysed efficiently under acidic conditions.^33^ For clinical applications, however, a specific challenge is that the degraded products of acetals are aldehydes. Although the molecular mechanisms of aldehyde toxicity are poorly understood, the toxicity of aldehydes is well documented and exposure poses a potential health risk.^48^ An elegant approach to bypass aldehyde formation is to vary the chemical structure through the use of ketals.^33^ Ketals show similar behaviour with respect to cleavability under acidic conditions, but as a degradation product they form ketones with a better safety profile. An alternative approach to creating pH‐cleavable materials is the incorporation of imine bonds, resulting from the dehydration reaction of an aldehyde or ketone with an amine. Similar to acetals and ketals, imines are relatively stable under neutral and basic conditions, but are prone to hydrolysis in acidic environments.^49^ Alternatively, hydrazones formed by hydrazine with aldehydes or ketones have also been utilized for the preparation of acid‐degradable networks. Hydrazone‐based networks are generally less sensitive to acid‐induced hydrolysis than their imine analogues.^49^ In addition to their higher stability, the degradation profile can be fine‐tuned by modifying the steric of hydrazone groups with alkyl linkers or incorporating hydroxylamine groups to facilitate gradual degradation in a targeted time window.^50^

While acid‐cleavable materials are thoroughly described in the literature,^19^, ^49^ there is only a limited number of examples based on base‐labile linkages. Commonly employed photoresists in DLW are multifunctional polyesters, such as PETA,^3^ poly(ethylene glycol) diacrylate,^4^, ^51^ pentaerythritol tetraacrylate,^52^ among others. Although polyesters can gradually degrade in harsh conditions, the required basic pH values are often impractical for many applications. One approach to reducing the necessary pH value is to activate the ester by incorporating electron‐withdrawing motifs, such as tetrafluorophenyl groups (Figure 3).^53^

## 4 Summary and Outlook

DLW is capable of fabricating complex 3D structures at very high resolution. In a short time, the field of smart microstructures with intrinsically programmed properties has developed from a niche to a vibrant research field. This Minireview provides a first overview of photoresist design for cleavable microstructures, including an illustration of the labile bonds, the corresponding cleavage conditions, and their potential application fields.

Overall, we foresee two major challenges that must be addressed by the research community. First, the toolbox of accessible cleavage triggers still needs to be expanded. In particular, biocompatible cleavage triggers, such as light, enzymes, and pH value, should be comprehensively investigated since they would be of great value in the fields of drug delivery, cell biology, and tissue engineering. These applications often additionally benefit from a slow and gradual degradation, which can be achieved by adjusting the photoresist structure accordingly. A downside of existing cleavable microstructure is that they often require elevated temperature or organic solvents for complete degradation. It remains to address these issues by seeking milder cleavage conditions that are biocompatible. Moreover, research is required to take full advantage of temperature‐cleavable microstructures. Specifically, research should not only focus on DA reactions between conjugated dienes and substituted alkenes, but also incorporate heteroatoms into the DA structure for obtaining cleavable materials within certain temperature windows. In addition to expanding the number of cleavage triggers, advanced approaches, such as orthogonal degradation, hold significant potential and need more research. We believe that combining different cleavage triggers, such as pH and light, or substrate‐specific enzymes, are promising to fill this gap.

Second, much of the latest research effort on cleavable direct‐laser‐written structures is focused on the incorporation of labile bonds into the photoresist structure, whereas little work has been carried out investigating the overall suitability for specific applications. For example, there are limited explorations of many key properties, such as feature resolution, toxicity of degraded products, or mechanical stability of the fabricated microstructures. This research is equally important to the photoresist design, because labile bonds might negatively affect other properties, which can make fabricated 3D structures unsuitable for desired applications.

Despite these challenges, cleavable direct‐laser‐written microstructures have great potential for various applications, which have been outlined. Given the recent pace in the field of degradable materials, we are optimistic that the obstacles and difficulties identified can be overcome and that on‐demand degradation of 3D microprints via DLW has a bright future ahead of it.

## Conflict of interest

The authors declare no conflict of interest.

## Biographical Information

*David Gräfe received his BSc (2010) and MSc (2012) in chemistry from the University of Technology Dresden (TUD). In 2017 he completed his PhD at the Leibniz Institute of Polymer Research Dresden (IPF) under the supervision of Prof. Brigitte Voit on polymeric materials for flow control in microfluidics. He is currently working with Prof. Barner‐Kowollik at the Queensland University of Technology (QUT) as a Fellow of the German Research Foundation. His research interests include the design and development of polymeric materials*, *in particular the polymer synthesis of responsive and adaptive materials*.

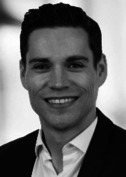

## Biographical Information

*Sarah L. Walden received a B.Math/BAppSc (Phys) (Hons) from the QUT, Brisbane, Australia, in 2012. She then went on to complete a PhD under the supervision of Prof. Esa Jaatinen investigating the nonlinear optical properties of semiconductor nanoparticles. Her research interests are in the area of light‐matter interactions and nonlinear optical properties of materials. She is currently a Postdoctoral Fellow on a joint project between the Soft Matter Materials Laboratory at QUT and the Wegener group at the Karlsruhe Institute of Technology (KIT), Germany, investigating new materials for sub‐diffraction resolution lithography*.

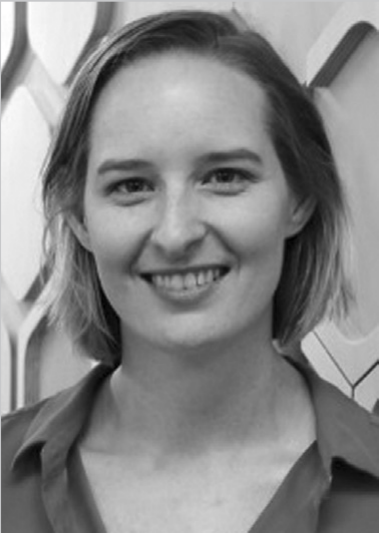

## Biographical Information

*James P. Blinco graduated from QUT, Australia, in 2009 with a PhD in organic chemistry under the supervision of Prof. S. Bottle. In 2009 he held a postdoctoral position working on polymer synthesis for next generation photoresists at the Centre for Magnetic Resonance (CMR) and the Australian Institute for Bioengineering and Nanotechnology (AIBN) at the University of Queensland. In 2010, he was awarded an Alexander von Humboldt research fellowship to work at the KIT with Prof Christopher Barner‐Kowollik. He is currently employed as a Senior Lecturer at QUT. His research interests include polymer synthesis, and free radical‐, electro and photo‐chemistry*.

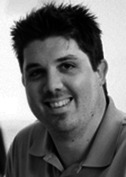

## Biographical Information

*Martin Wegener obtained his Diplom and his PhD in physics from the Johann Wolfgang Goethe‐Universität zu Frankfurt am Main in 1986 and 1987, respectively. After spending two years as a postdoctoral researcher at AT&T Bell Laboratories in Holmdel (USA), he became C3 professor at Dortmund University in 1990. In 1995 he became C4 professor at Institute of Applied Physics at what is now KIT. Since 2001, he has also been affiliated with the Institute of Nanotechnology at KIT. Since 2018, he is spokesperson of the Excellence Cluster “3D Matter Made to Order”, which is jointly carried by KIT and Heidelberg University*.

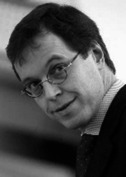

## Biographical Information

*Eva Blasco completed her PhD studies in 2013 at the University of Zaragoza (Spain) under the supervision of Prof. L. Oriol and Dr. M. Pinol. Thereafter, she obtained an Alexander von Humboldt Postdoctoral Research Fellowship to work in the groups of Prof. Barner‐Kowollik and Prof. Wegener (Applied Physics) at the KIT on the preparation of 3D conductive microstructures via 3D laser lithography. Since 2016 she has been working as a scientist at KIT. Her research interests include the development of new functional materials by employing light, particularly, for 3D laser lithography*.

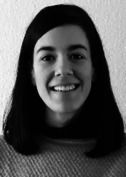

## Biographical Information

*Christopher Barner‐Kowollik received his PhD in 1999 from Göttingen University under the supervision of Prof. M. Buback. In 2006 he was appointed Full Professor of Polymer Chemistry at the Centre for Advanced Macromolecular Design at the University of New South Wales in Sydney. In 2008 he became chair for Macromolecular Chemistry at the KIT and is currently chair for Materials Chemistry at QUT and a group leader at the KIT. In 2016, he was awarded the Erwin‐Schroedinger Award of the Helmholtz Association (jointly with M. Wegener and M. Bastmeyer) and in 2017 a Laureate Fellowship by the Australian Research Council. He is a Fellow of the Australian Academy of Science*.